# 1,8-Cineole Potentiates the Antibacterial Activity of Amoxicillin/Clavulanic Acid Against an ESBL-Producing *Escherichia coli* Strain: An In Vitro and In Silico Investigation

**DOI:** 10.3390/ph19071094

**Published:** 2026-07-16

**Authors:** Mounia Oukhouia, Assia Houiat, Samira Oukhouia, Chaymae Moubachir, Mohd Yasir Khan, Farah Maarfi, Mohammed Cherkaoui, Adnane Remmal

**Affiliations:** 1 Department of Digital Engineering and Artificial Intelligence, College of Science, Long Island University, Brooklyn, NY 11201, USA; mounia.oukhouia@usmba.ac.ma (M.O.); mohd.yasirkhan@liu.edu (M.Y.K.); farah.maarfi@liu.edu (F.M.); mohammed.cherkaoui@liu.edu (M.C.); 2 Biotechnology, Environment, Agri-Food and Health Laboratory, Faculty of Sciences, Dhar El Mahraz, Sidi Mohamed Ben Abdellah University, P.O. Box 1796, Atlas, Fez 30050, Morocco; assia.houiat@usmba.ac.ma (A.H.); samira.oukhouia@usmba.ac.ma (S.O.); chaymae.moubachir@usmba.ac.ma (C.M.)

**Keywords:** amoxicillin, clavulanic acid, 1,8-cineole, resistance, synergy, molecular docking, breaking resistance, DFT

## Abstract

**Background/Objectives**: Antibiotic resistance in bacteria poses a major health problem worldwide. Therefore, to counteract this life-threatening problem, we sought to investigate in the present study the possible potentiation of the efficacy of amoxicillin (AMX) and clavulanic acid (CA) by 1,8-cineole (CN), a candidate resistance-modulating agent. The approach seeks to investigate, in vitro and in silico, the interactions among these three molecules. **Methods**: The antibacterial activity was determined against resistant *Escherichia coli* (*E. coli*) using microdilution methods, synergy tests, and time-kill assays for AMX, CA, and CN, used either separately or in combinations: AMX–CA, AMX–CN, CA–CN, and AMX–CA–CN. Furthermore, an in silico drug design methodology was employed, utilizing an integrated workflow that combines Density Functional Theory (DFT) for ligand optimization with molecular docking simulations to evaluate binding energies and interactions between the penicillin-binding protein (PBP) and ligands. **Results**: In vitro synergy experiments and time-kill assays revealed substantial antibacterial efficacy of the AMX–CA–CN combination. In silico analyses, performed under the simplifying assumption of a pre-assembled multi-ligand entity, were consistent with these findings: within our docking model, the AMX–CA–CN combination exhibited the most favorable computed binding affinity to a representative penicillin-binding protein (PBP3, PDB 7ONW). **Conclusions**: 1,8-cineole can potentiate the antibacterial effects of AMX–CA, indicating that the AMX–CA–CN combination warrants further evaluation as a candidate adjunctive strategy against ESBL-producing *E. coli*.

## 1. Introduction

The increasing prevalence of antibiotic-resistant bacteria poses a significant threat to global public health, undermining decades of medical advances and complicating treatment strategies [1]. Among these resistant pathogens, *E. coli* has emerged as a notable concern due to its widespread presence and its capacity to acquire and spread various resistance mechanisms [2].

Mutations in penicillin-binding proteins (PBPs) are the primary drivers of resistance to β-lactam antibiotics, which include penicillins, cephalosporins, and carbapenems. These mutations result in a diminished binding affinity for these drugs [3]. These structural shifts severely hinder the clinical application of β-lactams, although they remain the cornerstone of treatment for *E. coli* infections. Further, resistance is enhanced by the production of β-lactamases enzymes, which degrade the β-lactam ring and render the antibiotics ineffective [4].

PBPs are important targets for β-lactam antibiotics because they mediate the final steps of bacterial cell-wall synthesis. In *E. coli*, amoxicillin irreversibly binds to PBPs by acting like the natural substrate of peptidoglycan, which competitively inhibits the transpeptidation and the cell wall deformation [5]. This mechanism underscores the potential therapeutic value of β-lactam antibiotics in bacterial infections, wherein PBPs are critical for conserving the integrity of the bacteria [6,7].

While the discovery of new antibiotics is essential, their evolution is time-consuming, complex, and could take decades before such treatments become broadly available [8]. At the same time, some researchers are looking into more urgent and pragmatic remedies [9].

One possible strategy is to enhance the activity of existing antibiotics by combining them with alternative non-antibiotic drugs [10,11]. Notably, some currently approved medications for other medical uses have exhibited unexpected antibacterial properties [12]. Certain factors directly affect bacterial survival, while others affect antibiotic efficiency by lowering their MIC [13]. Such strategies offer a substantial opportunity to extend the duration of treatment efficacy of these antibiotics, serving as an adjunct to the development of new medications.

Our research explores the potential application of 1,8-cineole, a natural monoterpene oxide extracted from *Eucalyptus* essential oils, to break the resistance to β-lactam antibiotics [14]. While previous studies have documented 1,8-cineole’s antimicrobial and anti-inflammatory attributes, its impact on breaking bacterial resistance mechanisms has not been thoroughly examined [15].

We propose that 1,8-cineole may impact the interaction between amoxicillin, clavulanic acid, and PBP, potentially reversing the resistance of *E. coli* strains to β-lactam antibiotics. Our major aim in the present work is to introduce a novel adjunctive method to break the bacterial resistance to the widely used amoxicillin–clavulanic acid combination. The present study probes these hypotheses using complementary in vitro and in silico approaches, while acknowledging that the latter mechanisms cannot be excluded by the experiments reported here.

## 2. Results

### 2.1. In Vitro Studies

#### 2.1.1. MIC and MBC Determination

Table 1 displays the Minimum Inhibitory Concentration (MIC) and the Minimum Bactericidal Concentration (MBC) for AMX, CA, and CN when administered separately on *E. coli*. Table 2 illustrates the MIC and MBC values for the antimicrobials used in combination. Among the three, CN showed the highest MIC and MBC, indicating that a higher concentration is required to effectively inhibit bacterial growth, followed by AMX. On the other hand, CA exhibited the lowest MIC and MBC, suggesting that it represents the most potent antibiotic when used alone.

The MICs and MBCs for the antimicrobial combinations are outlined in Table 2, which shows that the triple combination (AMX, CA, CN) is the most effective with the lowest MIC and MBC, compared with values obtained when the components were added by pairs or separately (Table 1). This demonstrates strong antibacterial synergy between these three molecules. Indeed, the MIC of AMX was reduced from 2560 to 128 mg/L when paired with CN, and it was further decreased to 32 mg/L when combined with CA. A significant reduction was obtained for AMX when combined with CA and CN; the MIC value was reduced to 4 mg/L.

#### 2.1.2. Synergy Testing Using the Checkerboard Assay

The interactions among the antimicrobials were assessed using the Fractional Inhibitory Concentration (FIC) index, as shown in Table 3. The results indicate that the combinations of AMX, CA, and CN are synergistic, with the lowest FIC index value of 0.018 (Table 3). The FIC index values 0.075, 0.125, and 0.275 were obtained for the combinations AMX–CA, CA–CN, and AMX–CN, respectively (Table 3).

#### 2.1.3. Time-Kill Assay

**Figure 1:**
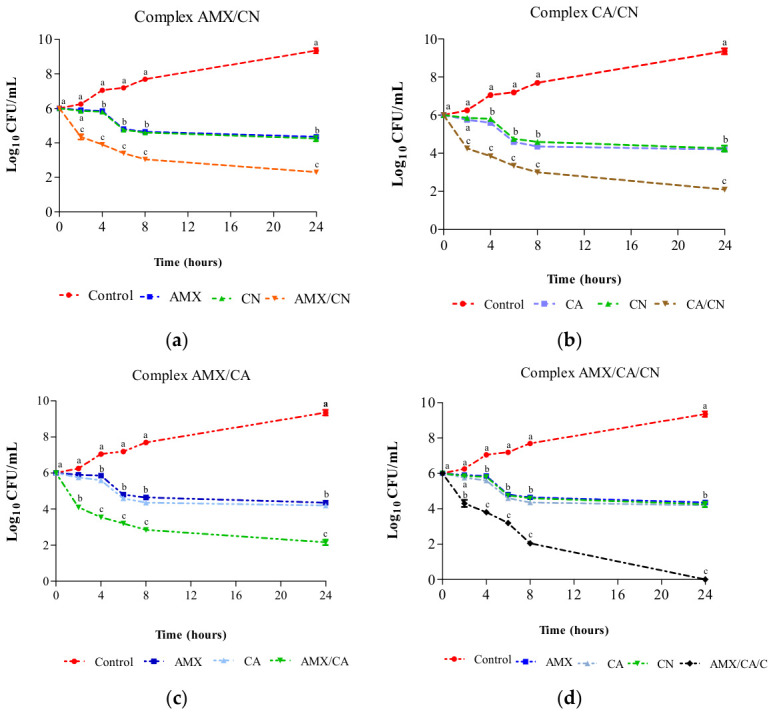
Bactericidal kinetics of AMX, CA, and CN alone and in combination against *E. coli*: (**a**) Bactericidal kinetic of AMX–CN; (**b**) Bactericidal kinetic of CA–CN; (**c**) Bactericidal kinetic of AMX–CA; (**d**) Bactericidal kinetic of AMX–CA–CN.

The bactericidal kinetics depicted in Figure 1 show that, when used separately, amoxicillin (MIC = 2560 mg/L), 1,8-cineole (MIC = 12,250 mg/L), and clavulanic acid (MIC = 128 mg/L) each reduced the colony counts by approximately 2 log units after 24 h. Nevertheless, when paired, each dual combination yielded an estimated 4-log reduction in bacterial populations. The combination of AMX, CA, and CN showed the most significant effect, resulting in a potent 6-log decrease in bacterial burden after 24 h of treatment.

### 2.2. In Silico Studies

#### 2.2.1. DFT Calculation Studies: Frontier Molecular Orbitals and Reactivity Descriptors

The energies of the highest occupied molecular orbitals (HOMOs) and the lowest unoccupied molecular orbitals (LUMOs) for the optimized ligands were determined using the Density Functional Theory (DFT) method (Figure S1) with the DMOL3 basis set, as shown in Table 4.

**Figure 2:**
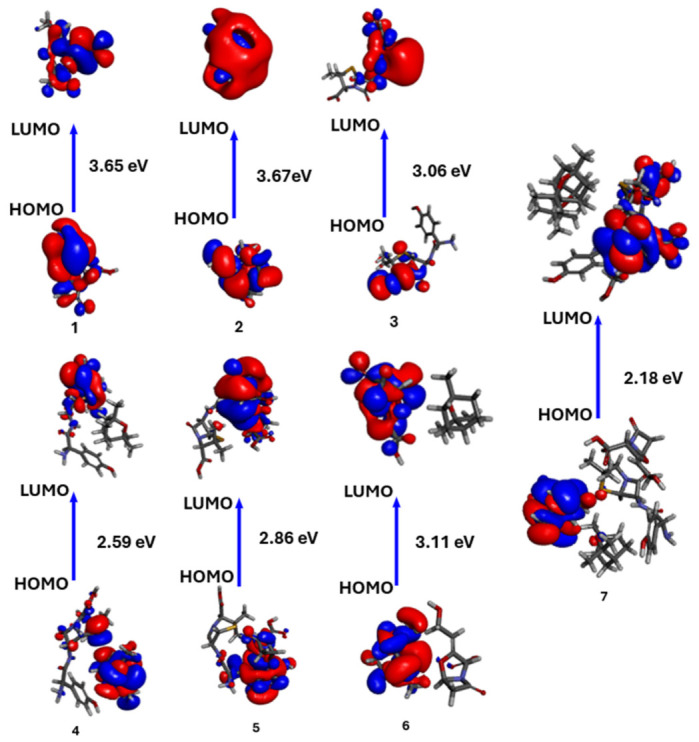
The calculated ground-state isodensity surface plots for Frontier molecular orbitals (FMOs) were generated using BIOVIA Discovery Studio, v. 24.1.0.321712. 1:CA, 2:CN, 3: AMX, 4: AMX–CN, 5: AMX–CA, 6: CA–CN, 7: AMX–CA–CN.

The isodensity surface plot for both single and multiple ligands, for HOMO and LUMO, is illustrated in Figure 2. This figure presents the spatial distribution of HOMO and LUMO for the optimized ligands. The HOMO indicates regions that are more prone to donating electrons, whereas the LUMO highlights areas that are likely to accept electrons. These orbitals serve as vital quantum-chemical parameters for assessing the chemical reactivity of molecules and are fundamental for calculating various significant descriptors of chemical reactivity. Based on the energy gap (∆E) among all examined compounds, they are ranked from most to least reactive as follows: AMX–CA–CN > AMX–CN > AMX–CA > AMX > CA–CN > CA > CN. The reactivity descriptors derived for all compounds (refer to Table 4) corroborate the findings indicated by the isodensity surface plots of HOMO and LUMO.

**Figure 3:**
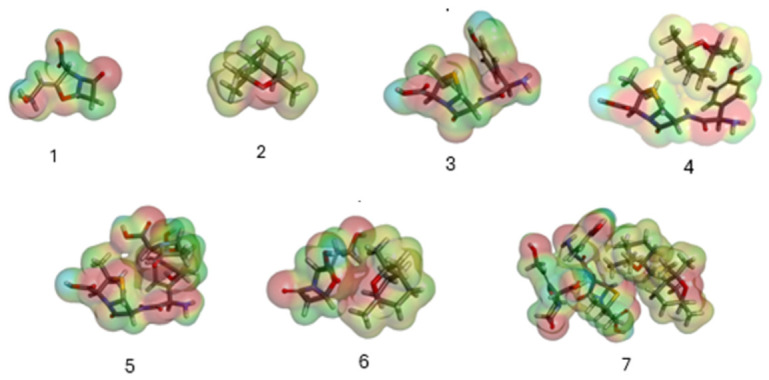
Molecular electrostatic potential (MEP) of all optimized ligands generated using BIOVIA Discovery Studio, v. 24.1.0.321712. 1:CA, 2:CN, 3:AMX, 4: AMX–CN, 5: AMX–CA, 6: CA–CN, 7: AMX–CA–CN.

All optimized ligands (1–7) were analyzed for their molecular electrostatic potential (MEP) as depicted in Figure 3. In this MEP analysis, regions with the highest negative charge are highlighted in red, indicating potential targets for electrophilic attack. Typically, these areas exhibit a negative charge that attracts electrophiles, while the blue regions indicate positively charged or neutral zones.

#### 2.2.2. Molecular Docking Simulation

**Figure 4:**
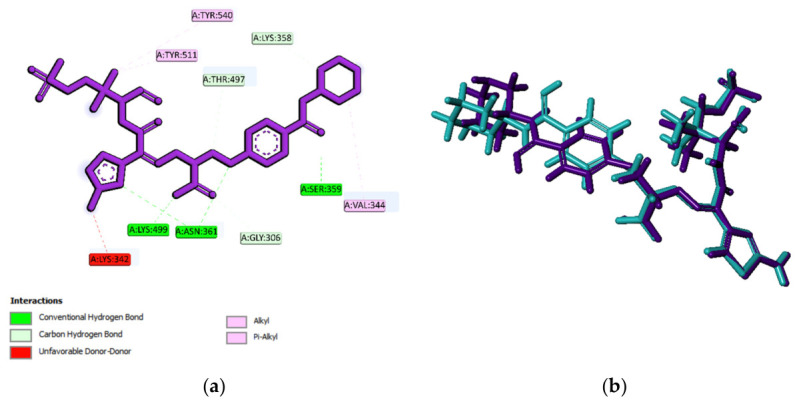
Results of redocking of the VL5 compound with the co-crystallized native VL5-7ONW complex. (**a**) 2D visualization of redocking of VL5 with target 7ONW. (**b**) The superimposition of the ligand position based on the redocking process of the VL5 compound with the co-crystallized VL5-7ONW complex (cyan blue: crystallography; purple: redocking).

To validate the further docking results of single and multi-ligands, the 7ONW receptor protein was re-docked with VL5. The redocking’s binding site area was x: 25.448756, y: 20.610622, and z: −23.826200, with a site sphere radius of 12.00. The RMSD analysis showed the degree of deviation between the experimental ligand docking results and the crystallographic ligand bound to the 7ONW protein at the same binding site. The redocking results indicated a 1.34114 Å RMSD value from the native ligand with the 7ONW receptor (Figure 4). Therefore, it can be concluded that the redocking method used in this study is valid and can be used against tested ligands with the same binding site area.

**Figure 5:**
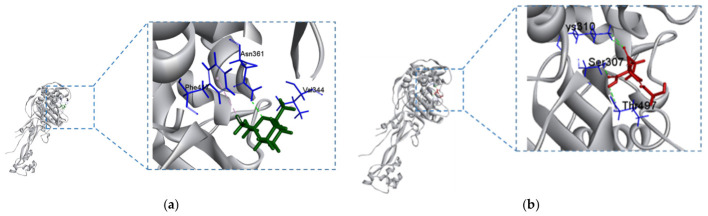

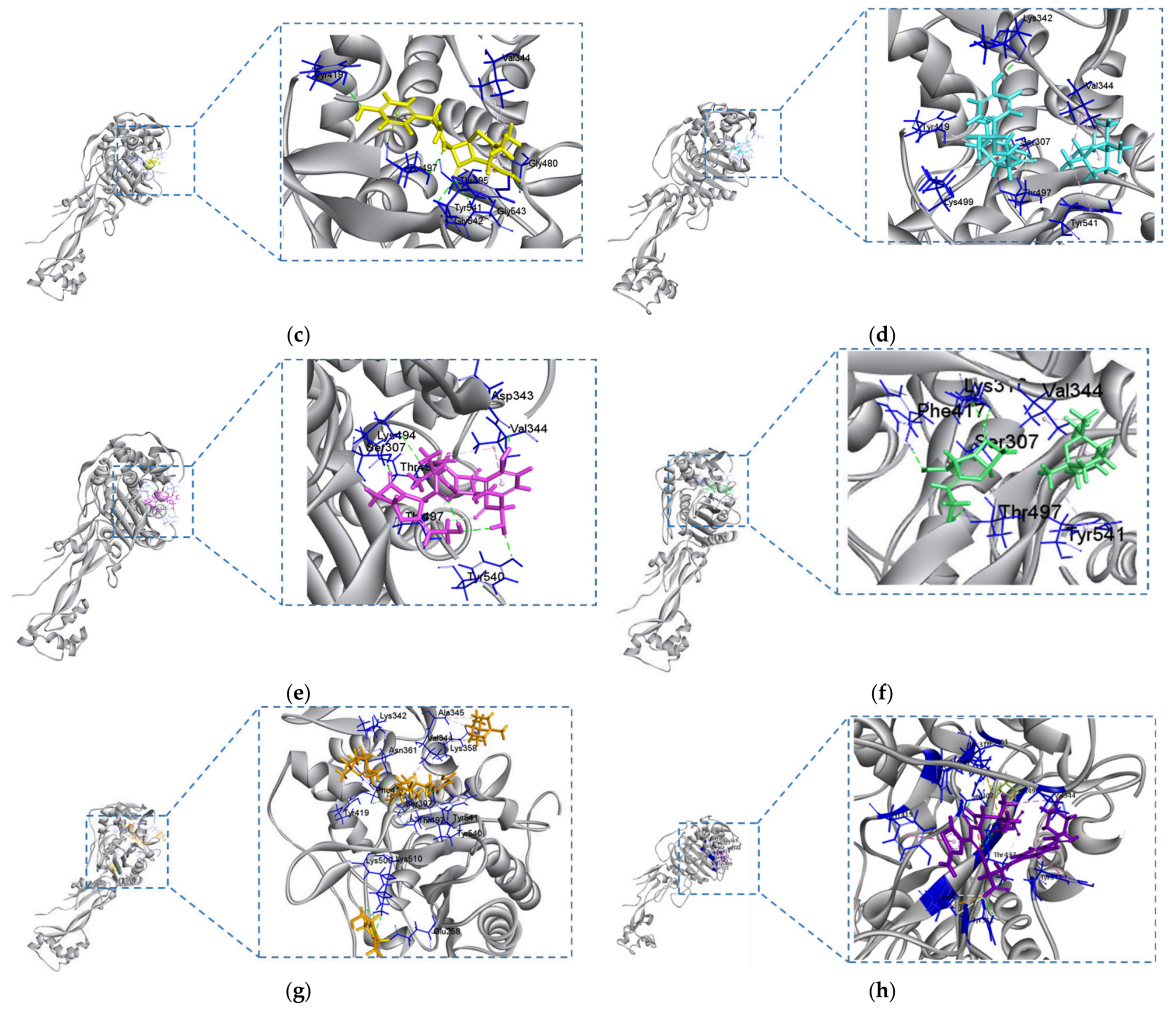
Summary of the docking poses of all optimized ligands with the crystal structure of 7NOW. (**a**) CN; (**b**) CA; (**c**) AMX; (**d**) AMX–CN; (**e**) AMX–CA; (**f**) CA–CN; (**g**) AMX–CA–CN; (**h**) VL5: co-crystalized inhibitor.

In our docking simulations against 7ONW, when the AMX–CA–CN combination was treated as a pre-assembled multi-ligand entity, it exhibited the most favorable computed binding affinity (−10.7 kcal/mol) and was predicted to engage seven key residues—Ser307, Val344, Asn361, Thr497, Tyr419, Tyr540, Tyr541—corresponding to those of the co-crystallized ligand (VL5). Conversely, the AMX–CA complex has an energy of −9.3 kcal/mol and binds only to five residues of the active site (Ser307, Val344, Lys494, Thr497, Tyr540), while AMX–CN (−9.1 kcal/mol) interacts with Ser307, Val344, Tyr419, Lys499, Tyr541. Finally, amoxicillin alone also binds to five residues (Val344, Tyr419, Thr495, Thr497, Tyr541) with an energy of −7.3 kcal/mol. These computational results suggest, within the limitations of our docking workflow, that the AMX–CA–CN entity has the most favorable predicted binding profile in terms of computed energy and the number of contacted residues (Table 5 and Figure 5).

## 3. Discussion

The present data expand upon our previous findings regarding the synergistic effects of amoxicillin and 1,8-cineole against hospital strains of *E. coli* and *Klebsiella pneumoniae* ESBL [16], as well as the combination of amoxicillin–clavulanic acid with 1,8-cineole on *Staphylococcus aureus* MRSA [17]. Multiple articles emphasize synergistic interactions between various kinds of antibiotics and essential oils or their constituents, including thymol, carvacrol, eugenol, and 1,8-cineole [18,19,20]. To our knowledge, the molecular mechanism elucidating this synergy remains ambiguous. The major aim of the present study was to contribute to a better understanding of the mechanism of action underlying the synergies observed among amoxicillin, clavulanic acid, and 1,8-cineole by examining molecular interactions between these three compounds using both in vitro and in silico approaches (Figures S1 and S3). This represents, to our knowledge, the first mechanistic investigation of the molecular interactions underlying 1,8-cineole β-lactam/β-lactamase inhibitor synergy. The novelty, therefore, lies not in the antibiotic’s known mode of action, but in elucidating how cineole enhances it at the molecular level.

According to the results shown in Table 1, the high MIC of the tested *E. coli* strain confirms its high resistance to amoxicillin alone; this resistance is due to the production of a CTX-M-type extended-spectrum β-lactamase, as reported by Arhoune et al. [21], who used the same strain. This isolate was selected as a representative, well-characterized clinical example of high-level β-lactam resistance driven by a CTX-M-type ESBL. This table also shows that clavulanic acid alone (without AMX) has significant antibacterial activity with a MIC 20 times lower than that of amoxicillin. This result indicates that clavulanic acid is not merely a β-lactamase inhibitor, as is commonly described [22,23], but it also possesses an antibiotic activity. This has already been reported as an intriguing finding by Finlay et al. [24] and by Higgins et al. [25] in β-lactamase-producing and non-β-lactamase-producing Gram-negative strains. The in silico results shown in Figure 5 and Table 5 demonstrate that clavulanic acid forms hydrogen bonds with the amino acids at the active site of the penicillin-binding protein (PBP) inhibitor [26]. This finding is consistent with the in vitro results obtained by Spratt et al. on *E. coli*, who demonstrated that clavulanic acid exerts its antibacterial action through the inhibition of PBP2 [27]. Consequently, it can be assumed that the well-known synergy between clavulanic acid and amoxicillin in β-lactamase-producing bacteria is due to the inhibition of both β-lactamase and PBP. This hypothesis was already elucidated by Servin et al. [26], who mentioned that CA enhances the antibacterial effect of AMX on non-β-lactamase producing Pneumococci. The synergistic interaction between AMX and CN may arise either from the individual activity of each molecule or from the formation of a molecular complex between them.

The results in Table 2 and Table 3 show that the combination of clavulanic acid (MIC × 1/8) and amoxicillin (MIC × 1/80) completely inhibits bacterial growth, with an FIC index of 0.075. Furthermore, the combination of AMX and CA at concentrations equal to the MIC of each showed a significant reduction in bacterial load of approximately 4-log during the bactericidal kinetics test, indicating synergy between the two compounds, whereas each compound alone induced only a 2-log reduction. The in silico results depicted in Figure 5 and Table 5 clearly show the presence of a hydrogen bond between these two compounds, which allows the formation of a molecular complex with high affinity for PBP. Geometric optimization of the ligands via DFT calculations, followed by molecular docking, confirms the formation of stable hydrogen bonds between amoxicillin and clavulanic acid when the complex is bound to PBP. These results are consistent with previous analytical chemistry studies that have demonstrated, using Differential Scanning Calorimetry (DSC), X-Ray Powder Diffraction (XRPD), Fourier-Transform Infrared Spectroscopy (FTIR), and Scanning Electron Microscopy (SEM), the presence of such bonds in the solid state [28]. A similar assumption was made by Navarro et al. [29], who noted that the pharmacokinetic variability observed when AMX is administered with CA is due to a possible interaction between the two molecules.

Regarding the synergy between the tested molecules, the results in Table 2 and Table 3 show that combining clavulanic acid at 1/4 of its MIC with 1,8-cineole at 1/40 of its MIC completely inhibits bacterial growth, with an FIC index of 0.275. In addition, results from the bactericidal kinetics test demonstrated that the combined use of CA and CN, each at its MIC, led to a significant decrease in bacterial load of approximately 4-log units, in contrast to the 2-log reduction observed with each compound individually. This enhanced killing effect indicates a synergistic interaction between CA and CN. A molecular docking study was performed to determine the binding affinity and interaction profile of the CA–CN complex. This complex exhibits a binding energy of −8.4 kcal/mol, indicating good affinity for the active site of penicillin-binding protein 7ONW. The study reveals six significant interactions with the residues Ser307, Lys310, Val344, Phe417, Thr497, and Tyr541. Among these, four residues (Ser307, Val344, Thr497, and Tyr541) coincide with those involved by the reference inhibitor VL5, indicating that the CA–CN complex forms a molecular complex and binds via four hydrogen bonds within the active pocket of the 7ONW structure.

Regarding the AMX–CN combination, in vitro results show a significant reduction in the MIC to 1/20 of AMX and 1/40 of CN, with a FIC index of 0.125. Notably, whereas AMX and CN alone reduced the bacterial load by only about 2-log units each, their combination at equivalent MICs achieved a significantly greater reduction of approximately 4-log units in the bactericidal kinetics assay, pointing to a synergistic relationship between the two compounds. These in vitro results are consistent with those obtained by Akhmouch et al. [16]. The molecular docking results for this combination, as shown in Figure 5 and Table 5, clearly demonstrate the affinity of the complex AMX–CN for the PBP 7ONW target used in this study. This affinity is reflected in strong hydrogen bonds involving the residues Ser 307, Asp 343, Val 344, Lys 494, Thr 495, Thr 497, and Tyr 540. The five amino acids Ser307, Val344, Lys494, Thr497, and Tyr540 are at the inhibitor’s active site with high binding energy. This prompted us to ask whether 1,8-cineole further potentiates the amoxicillin–clavulanic acid combination, which is a widely used antibiotic in clinical practice [30]. The in vitro results obtained with the AMX–CA–CN combination confirmed this hypothesis. Indeed, amoxicillin at 1/640 of its MIC, clavulanic acid at 1/60 of its MIC, and 1,8-cineole at 1/645 of its MIC are sufficient to completely halt bacterial growth with a very low FIC index of 0.018, indicating potent synergy among the three molecules. Notably, combining AMX, CA, and CN at their respective MIC values resulted in a substantial 6-log reduction in bacterial load during the kinetics assay, far exceeding the 2-log reduction observed with each compound alone, indicating strong synergy among the three agents. The synergistic effect observed with the triple combination of AMX, CA, and CN is reflected not only in a remarkably low FIC index but also in a clear restoration of bacterial susceptibility (CMI of AMX at 4 mg/L and CMI of CA at 2 mg/L). In comparison, the dual combinations (AMX–CA, AMX–CN, and CA–CN) also demonstrate synergistic interactions, as indicated by their FIC values, but failed to fully reverse resistance, as the bacteria remain classified as resistant according to the Clinical and Laboratory Standards Institute criteria [16].

The synergistic activity observed in this study was strongly concentration-dependent. Individual compounds at their MICs achieved only approximately 2-log CFU reductions, confirming insufficient bactericidal activity against the ESBL-producing strain. Binary combinations demonstrated full synergy by FIC criteria and approximately 4-log CFU reductions yet failed to restore clinical susceptibility. Only the ternary AMX–CA–CN combination achieved both complete growth inhibition (FIC = 0.018) and full reversal of resistance at clinical breakpoints. This outcome highlights an important distinction between FIC-defined synergy and clinically meaningful resistance restoration, two endpoints that are not equivalent and depend critically on the concentration ratios between interacting compounds. These findings suggest that the therapeutic benefit of the ternary combination lies in optimizing the relative concentrations of all three agents rather than increasing the dose of any single component.

A molecular docking analysis was also performed to evaluate the binding affinity and the interaction profile of the AMX–CA–CN complex, which exhibited the most significant interaction with the active site of PBP 7ONW, characterized by the highest binding affinity (−10.7 kcal/mol) and by fourteen amino acid residue among them seven notable interactions with residues Ser307, Val344, Asn361, Thr497, Tyr419, Tyr540, and Tyr541.

These residues correspond to those involved with the co-crystallized ligand (VL5) [31], suggesting the potential of this combination to effectively occupy the active pocket of 7ONW (Figure 5).

To further elucidate the relationship between the electronic properties and binding behavior of the studied ligands, the DFT-derived global electrophilicity index (ω) was correlated with the molecular docking binding energies across all tested compounds and combinations. Among the individual compounds, AMX displayed the highest electrophilicity index (ω = 4.32 eV) and the strongest binding affinity (−7.3 kcal/mol), followed by CA (ω = 4.24 eV; −6.3 kcal/mol) and CN (ω = 2.71 eV; −4.8 kcal/mol). This rank-order correspondence indicates that the electron-accepting capacity of individual compounds governs, at least in part, their propensity to interact favorably with the electron-rich nucleophilic residues lining the active site of PBP 7ONW. This trend was progressively amplified in the binary and ternary combinations: AMX–CN (ω = 5.42 eV; −9.1 kcal/mol), AMX–CA (ω = 4.21 eV; −9.3 kcal/mol), and CA–CN (ω = 3.72 eV; −8.4 kcal/mol) all exhibited enhanced binding affinities relative to their individual components, consistent with the emergence of electronic complementarity upon complexation. Strikingly, the ternary AMX–CA–CN complex achieved both the highest electrophilicity index (ω = 6.90 eV) and the most favorable binding energy (−10.7 kcal/mol) among all ligands evaluated, surpassing even the reference inhibitor VL5 (−7.9 kcal/mol).

The results suggest that the AMX–CA–CN complex exhibits greater stability and superior molecular recognition of 7ONW than the other combinations (AMX–CN, CA–CN, and AMX–CA) or the individual compounds (AMX, CA, and CN). The increase in the number of hydrogen bonds and higher binding energy indicates an improved thermodynamic profile and an enhanced fit with the enzyme’s catalytic domain. These interaction configurations suggest that the combined presence of CN and CA promotes a stronger binding of AMX to the penicillin-binding protein, which could enhance its ability to inhibit the latter’s enzymatic activity. Within the limits of our in silico methodology, these results support the working hypothesis that the AMX–CA–CN combination is the most promising of those evaluated in this study. Confirming this candidacy will require direct biophysical evidence of complex formation in solution, mechanistic experiments (e.g., β-lactamase activity assays and outer-membrane permeability tests), and validation across multiple resistant strains.

Our in vitro and in silico findings align with the clinical research conducted by Benjelloun et al. [32], which revealed that the combination of AMX, CA, and CN exhibited significant antibacterial effects while notably decreasing bacterial resistance in clinical environments. Furthermore, this strategy has shown favorable tolerability, suggesting that it may serve as a valuable and safe therapeutic option for treating respiratory infections.

Taken together, our in vitro and in silico observations are consistent with a working hypothesis that AMX, CA, and CN may combine to form various active entities, including dimers (AMX–CN, AMX–CA, CA–CN) and a ternary species (AMX–CA–CN) (Figure S1). We emphasize that this hypothesis requires direct experimental verification in solution, for example, by NMR, FTIR, isothermal titration calorimetry (ITC), or differential scanning calorimetry (DSC). If confirmed, such a configuration could plausibly exert multiple, simultaneous pressures on the bacterium, which might in turn slow the emergence of resistance.

## 4. Materials and Methods

### 4.1. In Vitro Studies

#### 4.1.1. Culture Media

Mueller–Hinton agar and broth (BIOKAR, Paris, France) were prepared and sterilized according to the manufacturer’s instructions.

#### 4.1.2. Bacterial Strain

The study utilized the ESBL *E. coli* strain, which was clinically isolated and provided by the Laboratory of Microbiology and Molecular Biology at the Faculty of Medicine and Pharmacy, Sidi Mohammed Ben Abdellah University, Fez, Morocco [21]. The inoculum suspension for the test was prepared by selecting colonies from 24 h cultures on MHA, which was then resuspended in sterile saline (0.9% NaCl). This strain was selected as a representative high-level β-lactam-resistant clinical isolate previously characterized as producing a CTX-M-type extended-spectrum β-lactamase, shaken for 15 s. The density was adjusted according to established optical density/concentration standard curves. The suspension was diluted in MHB to reach a final concentration of 3.3 × 10^6^ CFU/mL.

#### 4.1.3. Antimicrobial Agents

Amoxicillin trihydrate (AMX): Procured in powder form (CAS Number: 26787-78-0) from Sigma-Aldrich (Lyon, France), it was mixed until homogeneous and solubilized in sterile phosphate buffer (pH 6, 0.1 mol/L) to achieve a final concentration of 5120 mg/L.

Clavulanic Acid (CA): Obtained in powder form (CAS Number: 58001-44-8) from Sigma-Aldrich (Lyon, France), it was dissolved in sterile phosphate buffer (pH 6, 0.1 mol/L) and mixed thoroughly to yield a final concentration of 2560 mg/L.

1,8-cineol (CN): Purchased in liquid form (CAS Number: 470-82-6) from Sigma-Aldrich (Lyon, France), it was dispersed in 0.2% *w*/*v* agar in accordance with the procedure outlined by [33], resulting in a stock solution concentration of 125,000 mg/L.

#### 4.1.4. MIC and MBC Determination Assays

The minimum inhibitory concentration (MIC) and minimum bactericidal concentration (MBC) of the antimicrobials were established through a micro-dilution method in 96-well microplates, adhering to the CLSI 2024 standards, with some modifications [34]. Various volumes of three antimicrobials were added to the wells: AMX concentrations ranged from 2560 mg/L to 2 mg/L, CA concentrations from 256 mg/L to 0.5 mg/L, and CN concentrations from 25,000 mg/L to 48 mg/L. Bacterial suspensions, previously diluted in MHB, were inoculated into the 96-well microtiter plates to achieve a final density of 5 × 10^5^ CFU/mL. The plates were incubated at 37 °C for 18 to 24 h. Bacterial growth was assessed by adding 20 µL of a 0.5% (*w*/*v*) resazurin solution to each well. The MIC was defined as the lowest concentration showing no visible pink color after 2 h [35].

From wells exhibiting no apparent growth, a 15 μL aliquot is aseptically extracted and subsequently diluted tenfold prior to being inoculated onto MH agar to assess the MBC. The MBC is identified as the minimal concentration of the antibacterial agent that inhibits the observable growth of bacterial colonies on agar, as shown in Figure S3.

#### 4.1.5. Synergy Testing Using Checkerboard Assay

To evaluate the synergistic effects of the tested antimicrobials (AMX, CA, and CN), various combinations were prepared in sterile tubes in consistent dilutions. The antibacterial activity of these combinations was assessed using the microplate method. Each combination was conducted in triplicate, consisting of 50 µL of the diluted solution, 50 µL of the microorganism suspension (3.3 × 10^6^ CFU/mL), and 100 µL of MHB. A negative control was executed in triplicate by adding 100 µL sterile saline (0.9% NaCl) and 100 µL MHB to twelve wells horizontally. Three wells served as positive control, incorporating 100 µL of MHB, 50 µL of sterile saline (0.9% NaCl), and 50 µL of the bacterial suspension (3.3 × 10^6^ CFU/mL). Incubation was carried out at 37 °C for 18 to 24 h, with bacterial growth assessed via the addition of 20 µL of 0.5% water-soluble resazurin to each well. Synergy was defined as the lowest concentration that did not yield a pink color [35]. The interactions among the three antimicrobial agents were classified as synergistic, indifferent, or antagonistic based on their fractional inhibitory concentration index (FICI) values according to the following equation:

[ΣFICI = FIC(A) + FIC(B) + FIC(C)]

FIC (A) represents the ratio of the MIC (A) in combination to the MIC (A) alone, while FIC (B) is the ratio of the MIC (B) in combination to the MIC (B) alone. Additionally, FIC (C) is the ratio of the MIC (C) in combination to the MIC (C) alone. The FIC was interpreted as follows: FIC ≤ 0.5 indicates synergy; 0.5 < FIC ≤ 0.75 indicates partial synergy; 0.76 ≤ FIC ≤ 1.0 indicates additive effects; 1 < FIC ≤ 4 indicates no interaction (non-differential); and FIC > 4 indicates antagonism [36].

#### 4.1.6. Time-Kill Assay

Time-kill experiments were performed for AMX, CA, and CN, both individually and in combination, at their MICs. Overnight cultures of *E. coli* strains were diluted to an optical density of 0.1 at 600 nm and further diluted 100-fold, resulting in 3.3 × 10^6^ CFU/mL; moreover, 100 μL of antimicrobial dilutions was added. At designated time points (0, 2, 4, 6, 8, and 24 h), aliquots were taken and diluted using physiological saline solution. The dilutions were then plated onto MHA, and colony counts were determined after a 24 h incubation at 37 °C. A reduction in viable cell count of ≥2 log10 for the most effective single component was classified as synergy [36].

#### 4.1.7. Statistical Analysis

In vitro data were analyzed using One-Way ANOVA followed by Tukey’s multiple comparison test. Statistical analysis was conducted using GraphPad Prism (version 5.03), and results were expressed as mean values ± SEM (standard error of the mean). Differences of *p* ≤ 0.05 were considered statistically significant.

### 4.2. In Silico Studies

#### 4.2.1. Preparation and Structure Optimization of Ligands

The ligands utilized in this investigation were obtained in 3D-SDF format from the PubChem database. After energy minimization, the ligands underwent a minimization protocol for 10,000 steps using the CHARMm force field, followed by geometry optimization through DFT calculations using Dmol3 basis for both single and multiple ligands in BIOVIA Discovery Studio, v. 24.1.0.321712 [37].

#### 4.2.2. Chemical Reactivity Descriptors

The EHOMO and ELUMO are indicators for the prediction of the ionization potential (I = −EHOMO) and the electron affinity (A = −ELUMO) of molecules. In addition, the Frontier molecular orbitals are used in the estimation of other chemical reactivity descriptors such as electronegativity (χ), global hardness (η), softness (δ), and electrophilicity (ω). These are calculated according to the following equations:

(1) $$\chi =-\frac{1}{2}\left(E_{HOMO}+E_{LUMO}\right)$$

(2) $$\eta =-\frac{1}{2}\left(E_{HOMO}-E_{LUMO}\right)$$

(3) $$\delta =\frac{1}{\eta }$$

(4) $$\omega =\frac{\chi^{2}}{2\eta }$$

The χ value is a prediction of the power of the molecule to attract electrons, while small values of (χ) are an indication of a good base. The global hardness (η) is a degree of their charge transfer prohibition; however, the global softness (δ) characterizes the ability of a molecule to accept electrons. The complexation energy was also calculated (Table S1).

#### 4.2.3. Proteins Preparation

The protein employed in this research was co-crystallized, and its 3D structure (as shown in Table 6) was retrieved from the RCSB Protein Data Bank (PDB). This structure was optimized using the prepared protein protocol within BIOVIA Discovery Studio, v. 24.1.0.321712, applying the CHARMm force field [38].

#### 4.2.4. Redocking to Confirm the Interaction Between the Target Protein and Ligands

To confirm that ligands can dock to the target protein, the PBP receptor (PDB ID:7ONW) was docked with its co-crystallized ligand inhibitor VL5. If the RMSD value is ≤2 Å, the method is considered valid. This indicates that the test compound can be docked with the target protein in the same radius of the sphere site [39].

#### 4.2.5. Molecular Docking of Single Ligands and Multi-Ligands Against the Target Protein

A receptor grid was established around the binding pocket of the 7ONW protein, emphasizing critical active amino acid residues before docking single and multiple ligands. Ligands associated with the protein were chosen to identify amino acid residues that could serve as predictors for the binding site. The sphere delineating the binding site of the produced receptor grid box was defined by its x, y, z coordinates and radius, as detailed in Table 7.

For each ligand, ten docking poses were conventionally produced with the CDOCKER docking methodology. Molecular docking was employed to visualize ligand-target interactions and identify compounds with improved binding affinity using the BIOVIA Discovery Studio tool. The docking study encompassed docking scores, an assessment of ligand-protein interactions with specific emphasis on hydrogen bonds, and the imaging of docked complexes. The binding free energy of the protein-ligand complexes was calculated using the MM–GBSA approach, which combines molecular mechanics (MM) with the Generalized Born (GB) continuum solvation model and incorporates a surface area (SA) nonpolar solvation term [40].

## 5. Conclusions

This study demonstrates in vitro that 1,8-cineole potentiates the antibacterial activity of the amoxicillin/clavulanic acid combination against resistant ESBL-producing *E. coli* through a synergistic mechanism, supported by complementary in vitro and in silico approaches. Collectively, these findings provide hypothetical mechanistic evidence that 1,8-cineole acts as a resistance-breaking adjuvant by stabilizing a ternary molecular complex with enhanced affinity for the PBP active site, thereby overcoming β-lactam resistance in ESBL-producing *E. coli*. The AMX–CA–CN combination thus represents a candidate for the development of broad-spectrum antibacterial agents against resistant Gram-negative pathogens. Future research should focus on evaluating the in vivo efficacy and safety profiles of these molecular complexes, along with comprehensive mechanistic studies to elucidate the specific molecular pathways through which they exert their antibacterial effects.

## 6. Patents

Pharmaceutical Formulation Comprising 1,8-cineole and Amoxicillin (2019). Available online: https://patentscope.wipo.int/search/en/detail.jsf?docId=US250863563&docAn=16306262 (accessed on 1 April 2026).

## Figures and Tables

**Table 1:** MIC and MBC values of AMX, CA, and CN alone.

	AMX	CA	CN
MIC mg/L	2560	128	12,250
MBC mg/L	>2560	256	12,250

**Table 2:** MIC and MBC values of AMX, CA, and CN in combination.

	AMX + CN		CA + CN		AMX + CA		AMX + CA+ CN		
	AMX	CN	CA	CN	AMX	CA	AMX	CA	CN
MIC mg/L	128	312.5	32	312.5	32	16	4	2	19
MBC mg/L	128	312.5	32	312.5	64	32	4	2	19

**Table 3:** FIC Index values for the different combinations of *E. coli*.

MIC Alone (mg/L)			MIC Combined (mg/L)			FIC Index	Result
AMX	CA	CN	AMX	CA	CN		
2560	128	12,250	128	-	312.5	0.125	Synergy
2560	128	12,250	-	32	312.5	0.275	Synergy
2560	128	12,250	32	16	-	0.075	Synergy
2560	128	12,250	4	2	19	0.018	Synergy

**Table 4:** Calculated electronegativity (χ), global hardness (η), softness (δ), global electrophilicity index (ω), the ionization potential (I), and the electron affinity (A) (in eV).

	E_HOMO_	E_LUMO_	∆E	*Χ*	*H*	Δ	ω	Ι	A
CA	−5.76	−2.11	3.65	3.93	1.82	0.54	4.24	5.76	2.11
CN	−4.99	−1.32	3.67	3.15	1.83	0.54	2.71	4.99	1.32
AMX	−5.15	−2.14	3.06	3.64	1.53	0.65	4.32	5.15	2.14
AMX–CN	−5.00	−2.48	2.59	3.74	1.29	0.77	5.42	5.00	2.48
AMX–CA	−4.90	−2.04	2.86	3.47	1.43	0.69	4.21	4.90	2.04
CA–CN	−4.96	−1.85	3.11	3.40	1.55	0.64	3.72	4.96	1.85
AMX–CA–CN	−4.97	−2.79	2.18	3.88	1.09	0.91	6.90	4.97	2.79

**Table 5:** Summary of the interacting patterns of all optimized ligands with the crystal structure of 7ONW.

Ligand	Binding Energy (Kcal/mol)	MMGBSA (Kcal/mol)	-CDOCK Energy (Kcal/mol)	CDOCK Interaction Energy	Key Amino Acid Residue	Number of H-Bonds
CA	−6.3	−58.92	−18.26	25.30	Ser307 Ser35Lys494 Thr497	4
CN	−4.8	−24.19	−10.95	16.23	Val344 Asn361 Phe417	1
AMX	−7.3	−62.09	−24.57	42.87	Val344 Tyr419 Gly480 Thr495 Thr497 Tyr541 Gly542 Gly543	2
AMX–CN	−9.1	−78.02	−12.91	53.65	Ser307 Lys342 Val344 Tyr419 Thr497 Lys499 Tyr541	4
AMX–CA	−9.3	−95.33	−10.37	61.73	Ser 307 Asp343 Val344 Lys494 Thr495 Thr497 Tyr540	5
CA–CN	−8.4	−73.66	−18.87	50.87	Ser307 Lys310 Val344 Phe417 Thr497 Tyr541	4
AMX–CA–CN	−10.7	−128.78	−39.65	74.55	Glu258 Ser307 Lys342 Val344 Ala345 Lys358 Asn361 Phe417 Tyr419 Thr497 Lys500 Lys510 Tyr540 Tyr541	5
VL5	−7.9	−75.45	−28.42	49.38	Ser307 Lys310 Val344 Asn361 Phe417 Tyr419 Lys494 Gly496 Lys499 Thr497 Tyr511 Tyr541	4

**Table 6:** Protein information.

Protein	PDB ID	Resolution (Å)	Organism
Penicillin-binding protein	7ONW	2.70	*E. coli*

**Table 7:** Binding site coordinates of selected targets.

PDB ID	Binding Site Sphere (x, y, z)	Radius
7ONW	25.448756, 20.610622, −23.826200	12.000000

## Data Availability

The data used to support the findings of this study are available from the corresponding author upon request.

## Supplementary Materials

The following supporting information can be downloaded at: https://www.mdpi.com/article/10.3390/ph19071094/s1. Figure S1: Optimized structure of all tested compounds. (a) Clavulanic acid; (b) amoxicillin; (c) 1,8-cineole; (d) amoxicillin-1,8-cineole; (e) clavulanic acid-1,8-cineole; (f) amoxicillin–clavulanic acid; (g) amoxicillin–clavulanic acid-1,8-cineole. Figure S2: Representation of the molecular interaction of all optimized ligands in the binding sites of the structure of PBP 7ONW. (a) CN; (b) CA; (c) AMX; (d) AMX–CN; (e) AMX–CA; (f) CA–CN; (g) AMX–CA–CN. Figure S3: Schematic steps to determine MIC and MBC values. Table S1: Calculated complexation energy of all tested compounds. Table S2: Summary of the interacting patterns (key amino acid residue and type of interactions) of all optimized ligands with the crystal structure of 7ONW.

## Abbreviations

The following abbreviations are used in this manuscript:

AMX	Amoxicillin
CA	Clavulanic acid
CN	1,8-cineole
MIC	Minimal inhibitory concentration
MBC	Minimal bactericidal concentration
DFT	Density Functional Theory
FICI	Fractional inhibitory concentration index
MEP	Molecular electrostatic potential
PBP	Penicillin-binding protein
