# Supplementary material for: 1,8-Cineole Potentiates the Antibacterial Activity of Amoxicillin/Clavulanic Acid Against an ESBL-Producing Escherichia coli Strain: An In Vitro and In Silico Investigation
Source: Pharmaceuticals (Basel). 2026 Jul 16;19(7):1094. doi: 10.3390/ph19071094 (PMC13414517; doi:10.3390/ph19071094)
Supplement: Supplementary file 1 [file pharmaceuticals-19-01094-s001.zip › pharmaceuticals-4382544-supplementary.pdf]

Supplementary Material

1,8-Cineole Potentiates the Antibacterial Activity of Amoxicillin/Clavulanic Acid Against an ESBL-Producing *Escherichia coli* Strain: An In Vitro and In Silico Investigation

Mounia Oukhouia [1,2], Assia Houiat [2], Samira Oukhouia [2], Chaymae Moubachir [2], Mohd Yasir Khan [1], Farah Maarfi [1], Mohammed Cherkaoui [1] and Adnane Remmal [2,*]

[1] Department of Digital Engineering and Artificial Intelligence, College of Science, Long Island University, Brooklyn, NY 11201, USA
[2] Biotechnology, Environment, Agri-Food and Health Laboratory, Faculty of Sciences, Dhar El Mahraz, Sidi Mohamed Ben Abdellah University, P.O. Box 1796, Atlas, Fez 30050, Morocco;
* Correspondence: adnaneremmal@gmail.com

Table of Contents

Figure S1: Optimized structure of all tested compounds. [a]: CA; [b]: AMX; [c]: CN; [d]: AMX-CN; [e]: CA-CN; [f]: AMX-CA; [g]:AMX-CA-CN.

Figure S2 : Representation of the molecular interaction of all optimized ligands in the binding sites of the structure of PBP 7ONW. (a): CN (b) CA; (c): AMX (d): AMX-CN; (e): AMX-CA; (f): CA-CN; (g): AMX-CA-CN

Figure S3: Schematic steps to determine MIC and MBC values.

Table S1: Calculated complexation energy of all tested compounds

Table S2: Summary of the interacting patterns (key amino acid residue and type of interactions) of all optimized ligands with crystal structure of 7ONW.

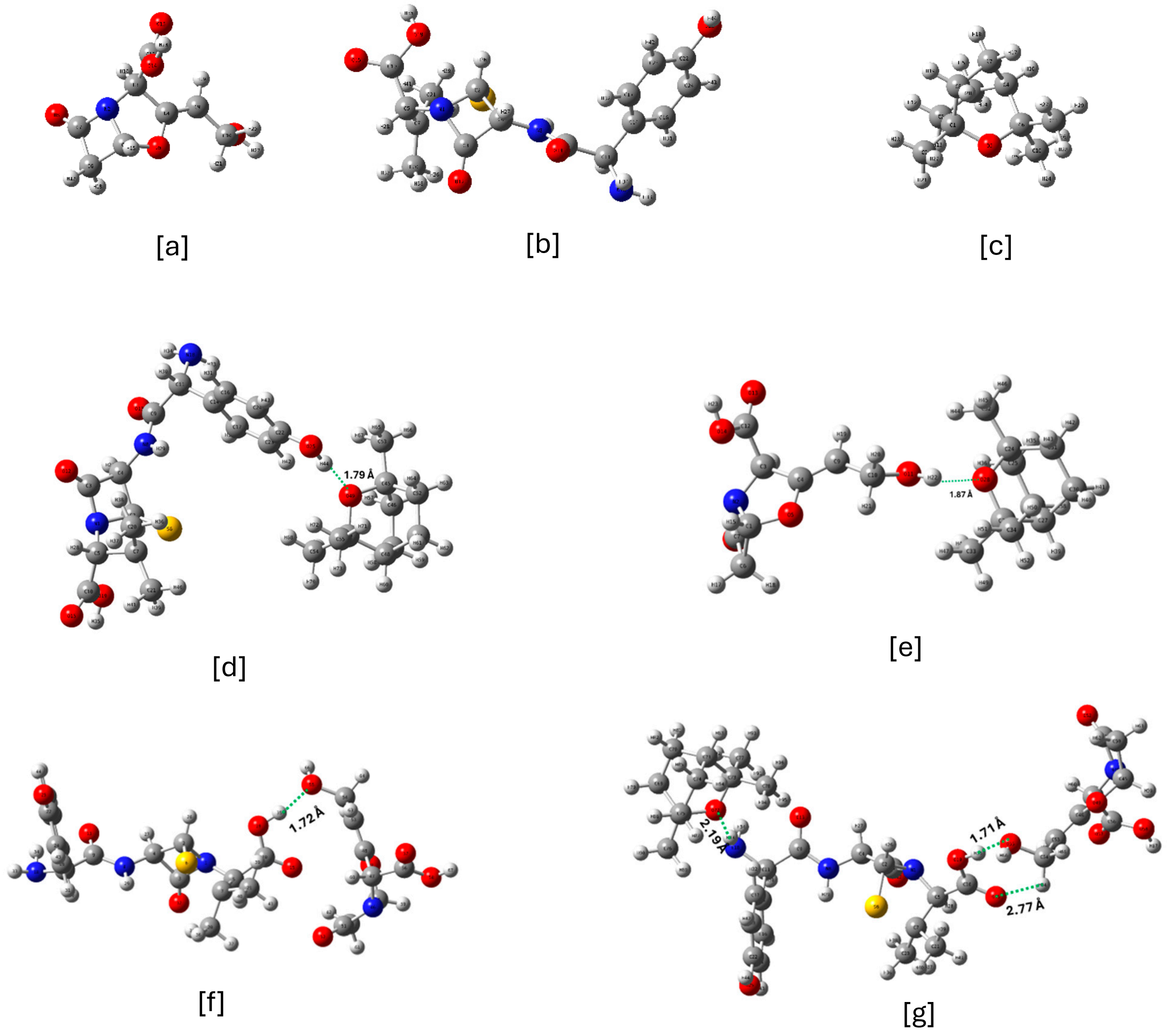

Figure S1. Optimized structure of all tested compounds. [a]: CA; [b]: AMX; [c]: CN; [d]: AMX-CN; [e]: CA-CN; [f]: AMX-CA; [g]:AMX-CA-CN.

Table S1. Calculated complexation energy of all tested compounds.

	**Complexation Energy Kcal/mol**
**CA**	-
**CN**	-
**AMX**	-
**AMX/CN**	-11.4
**AMX/CA**	-15.2
**CA/CN**	-9.10
**AMX/CA/CN**	-18.7

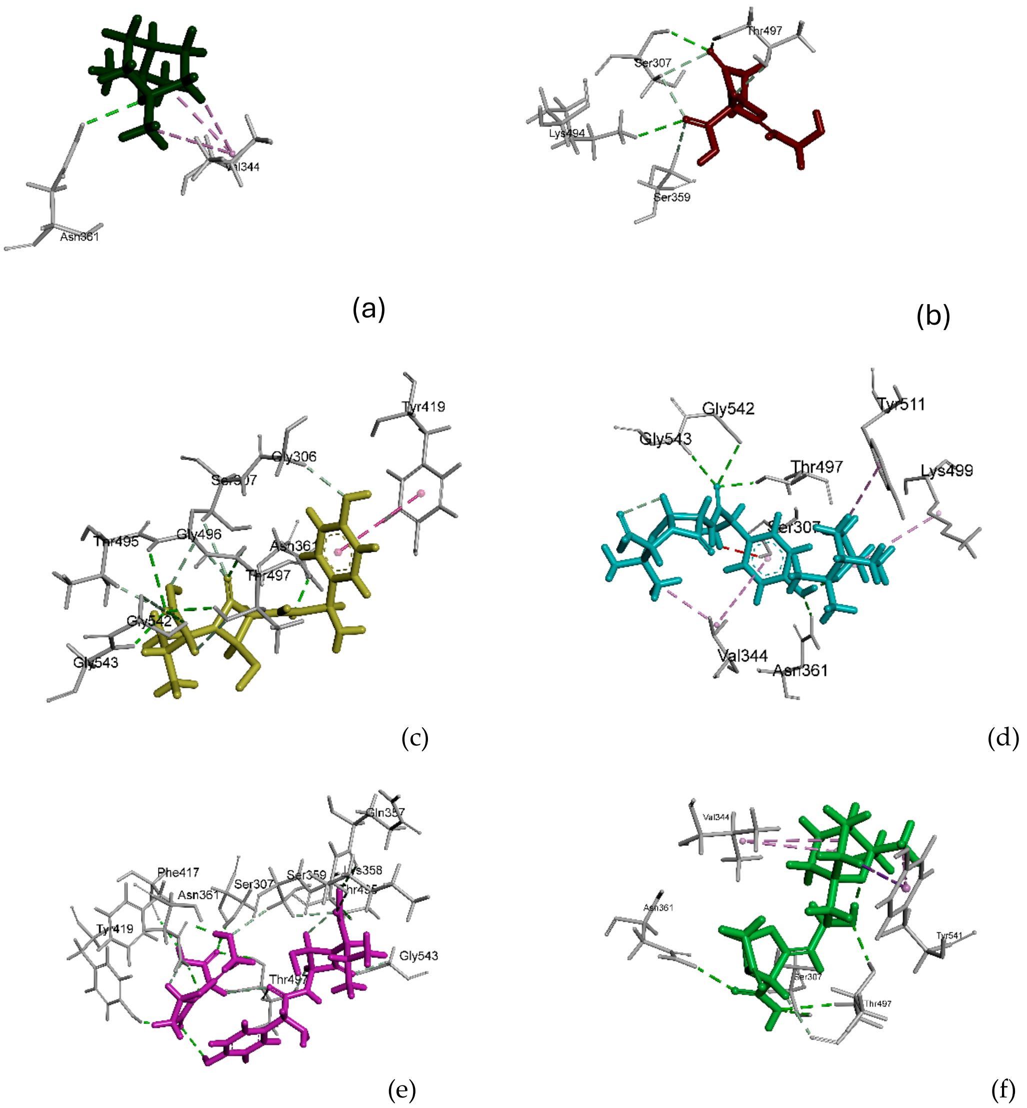
Asn361
(a)
Thr497
Ser307
Lys494
Ser359
(b)
Tyr419
Gly306
Thr495
Gly496
Asn361
Thr497
Gly542
Gly543
(c)
Gly542
Gly543
Tyr511
Thr497
Lys499
Val344
Asn361
(d)
Gln357
Phe417
Ser307
Ser359
Lys358
Asn361
Tyr419
Gly543
(e)
Val344
Asn361
Tyr541
Ser307
Thr497
(f)

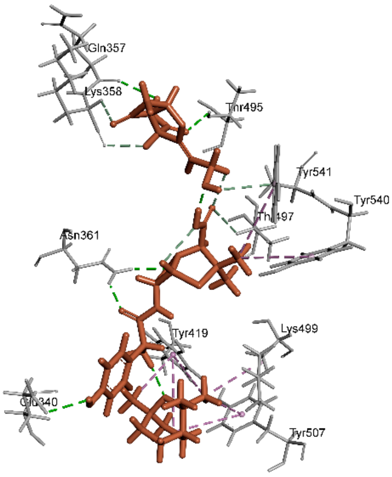

(g)

Figure S2 . Representation of the molecular interaction of all optimized ligands in the binding sites of the structure of PBP 7ONW. (a): CN (b) CA; (c): AMX (d): AMX-CN; (e): AMX-CA; (f): CA-CN; (g): AMX-CA-CN.

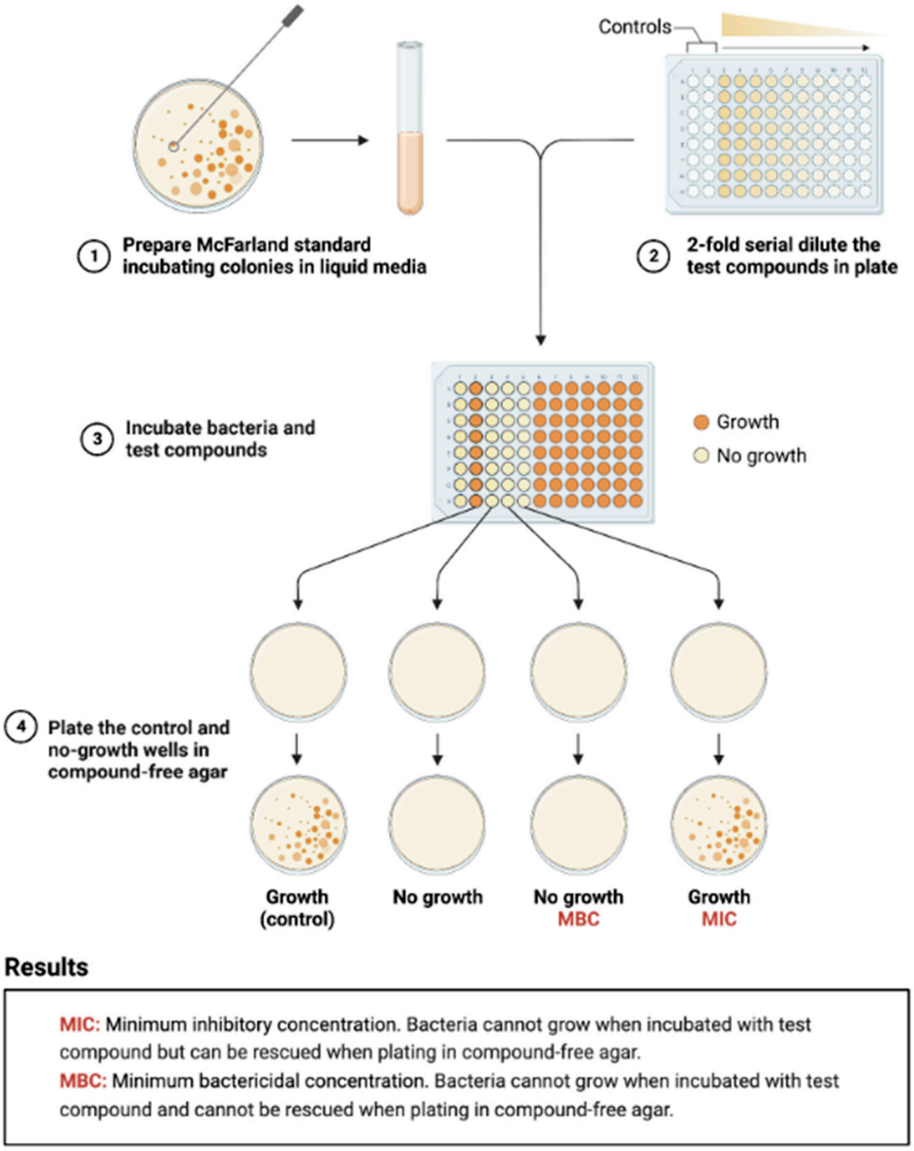

Figure S3. Schematic steps to determine MIC and MBC values.

Table S2. Summary of the interacting patterns (key amino acid residue and type of interactions) of all optimized ligands with crystal structure of 7ONW.

Ligand	Key amino acid residue	Type of interaction
**CA**	Ser307 Ser35Lys494 Thr497	Conventional hydrogen Bond
**CN**	Val344 Asn361 Phe417	Carbon hydrogen bond, Alkyl
**AMX**	Val344 Tyr419 Gly480 Thr495 Thr497 Tyr541 Gly542 Gly543	Conventional hydrogen Bond
**AMX-CN**	Ser307 Lys342 Val344 Tyr419 Thr497 Lys499 Tyr541	Conventional hydrogen Bond, Carbon hydrogen bond, Alkyl, Pi-Alkyl
**AMX-CA**	Ser 307 Asp343 Val344 Lys494 Thr495 Thr497 Tyr540	Conventional hydrogen Bond, Carbon hydrogen bond, Alkyl, Pi-Alkyl
**CA-CN**	Ser307 Lys310 Val344 Phe417 Thr497 Tyr541	Conventional hydrogen Bond, Carbon hydrogen bond, Alkyl, Pi-Alkyl
**AMX-CA-CN**	Glu258 Ser307 Lys342 Val344 Ala345 Lys358 Asn361 Phe417 Tyr419 Thr497 Lys500 Lys510 Tyr540 Tyr541	Conventional hydrogen Bond, Carbon hydrogen bond, Alkyl, Pi-Alkyl
**VL5**	Ser307 Lys310 Val344 Asn361 Phe417 Tyr419 Lys494 Gly496 Lys499 Thr497 Tyr511 Tyr541	Conventional hydrogen Bond, Carbon hydrogen bond, Alkyl, Pi-Alkyl, unfavorable bond
